# Conserved genomic organisation of Group B Sox genes in insects.

**DOI:** 10.1186/1471-2156-6-26

**Published:** 2005-05-19

**Authors:** Carol McKimmie, Gertrud Woerfel, Steven Russell

**Affiliations:** 1 Department of Genetics, University of Cambridge, Downing Street, Cambridge, CB2 3EH, UK

## Abstract

**Background:**

*Sox *domain containing genes are important metazoan transcriptional regulators implicated in a wide rage of developmental processes. The vertebrate B subgroup contains the *Sox1*, *Sox2 and Sox3 *genes that have early functions in neural development. Previous studies show that *Drosophila *Group B genes have been functionally conserved since they play essential roles in early neural specification and mutations in the *Drosophila Dichaete *and *SoxN *genes can be rescued with mammalian *Sox *genes. Despite their importance, the extent and organisation of the Group B family in *Drosophila *has not been fully characterised, an important step in using *Drosophila *to examine conserved aspects of Group B *Sox *gene function.

**Results:**

We have used the directed cDNA sequencing along with the output from the publicly-available genome sequencing projects to examine the structure of Group B *Sox *domain genes in *Drosophila melanogaster*, *Drosophila pseudoobscura, Anopheles gambiae *and *Apis mellifora*. All of the insect genomes contain four genes encoding Group B proteins, two of which are intronless, as is the case with vertebrate group B genes. As has been previously reported and unusually for Group B genes, two of the insect group B genes, *Sox21a *and *Sox21b*, contain introns within their DNA-binding domains. We find that the highly unusual multi-exon structure of the *Sox21b *gene is common to the insects. In addition, we find that three of the group B *Sox *genes are organised in a linked cluster in the insect genomes. By *in situ *hybridisation we show that the pattern of expression of each of the four group B genes during embryogenesis is conserved between *D. melanogaster *and *D. pseudoobscura*.

**Conclusion:**

The DNA-binding domain sequences and genomic organisation of the group B genes have been conserved over 300 My of evolution since the last common ancestor of the Hymenoptera and the Diptera. Our analysis suggests insects have two Group B1 genes, *SoxN *and *Dichaete*, and two Group B2 genes. The genomic organisation of *Dichaete *and another two Group B genes in a cluster, suggests they may be under concerted regulatory control. Our analysis suggests a simple model for the evolution of group B Sox genes in insects that differs from the proposed evolution of vertebrate Group B genes.

## Background

The family of Sox-domain containing proteins encompass a group of metazoan transcriptional regulators first identified by their similarity with the mammalian testis-determining factor SRY. Membership of the Sox family is conferred by the presence of an HMG1-type DNA-binding domain sharing greater than 60% amino-acid sequence identity to that of SRY [1]. Mammalian genome sequencing projects indicate that in humans and mice there are twenty *Sox *genes [2], divided into eight subgroups (A-H) on the basis of sequence identity within and outwith the HMG-domain. Aside from mammals, *Sox *genes have been identified in all metazoans examined to date, including birds, fish amphibians, basal chordates, insects and nematodes [3].

The B subgroup is of particular interest since members of this group are most closely related to SRY and appear to be functionally conserved during evolution. Sequence analysis and functional studies suggest that, in vertebrates, the five members of the B subgroup can be subdivided into two further groups; B1; *Sox1*, *Sox2 *and *Sox3*; [4] and B2; *Sox14 *and *Sox21*; [5]. It has been suggested from studies in the chick that the three group B1 proteins act as gene activators whereas the B2 proteins act as gene repressors [6]. In terms of genomic organization, all five of the group B genes are devoid of introns. *Sox3 *is located on the mammalian *X *chromosome and is believed to be the ancestor of *Sry *[7,8]. In humans, the remaining four autosomal group B genes are arranged in two pairs, each comprising one B1 gene and one B2 gene: *Sox2 *and *Sox14 *map together on chromosome 3 [9,10] and *Sox1 *and *Sox21 *map together on chromosome 13 [5,11]. This organization is conserved, at least in part, in other vertebrates with *Sox2-Sox14 *mapping together in the chick and the monotreme, *O. anatinus*, and *Sox1*-*Sox21 *mapping together in the chick [12,13]. There is, however, no linkage of Group B *Sox *genes in the mouse genome [14,15]. A model suggesting the evolution of group B genes and *Sry *from a single ancestor has been proposed, which suggests that pairs of B1 and B2 genes arose by a tandem duplication and then a chromosomal duplication [13].

The fruitfly, *Drosophila melanogaster*, has proved to be a tractable system for studying conserved aspects of eukaryotic gene function and, with the production of other insect genome sequences, a useful baseline for evolutionary studies of gene organisation [16]. Whole-genome sequence is now available for three insects, *Drosophila melanogaster, Drosophila pseudoobscura *(which diverged from *melanogaster *some 46 million years ago) and *Anopheles gambiae*, which diverged from *melanogaster *approximately 250 million years ago [17,18]. Sequencing and assembly of a further ten *Drosophila *species is currently underway [19] promising an unparalleled data source for evolutionary studies. In addition to the diptera, the sequencing of the Hymenoptera, *Apis mellifera *(honey bee ~280 million years from *Drosophila*), is now well underway, allowing fragments of a fourth insect genome to be assessed. In functional terms, *Drosophila *is a useful model for studying *SOX *gene function due to its genetic tractability. For example, we have previously shown that, in the case of the *Drosophila *group B gene *Dichaete*, there is functional conservation between insect and mammalian genes [20]. In addition, we, and others, have demonstrated a degree of *in vivo *functional redundancy between *Dichaete *and *SoxN *[21,22] as had been proposed for the mammalian group B genes [23]. Of particular interest is the fact that the expression patterns and functional studies of group B genes suggest that they participate in the earliest events of CNS differentiation in all organisms that have been studied to date including *Drosophila*, *Xenopus*, chick, mouse, ascidians and hemichordates [24].

To further explore the relationship between group B *Sox *genes we examined the extent and organization of the family in insects. Our studies show that group B *Sox *gene organisation is similar in four different insects. We find conservation in the sequence and genome organization of the group B genes in *D. melanogaster*, *D. pseudoobscura*, *A. gambiae *and *A. melifora*. In contrast to mammals and in agreement with a previous report [25], we find that two group B2 genes contain introns and are organized as a single genomic cluster along with the intronless *Dichaete *gene. Our studies indicate a potentially different evolutionary path for members of the group B family in insects and vertebrates.

## Results

To explore the structure of the group B *Sox *genes in insects we first accurately determined the extent and structure of the family in *Drosophila melanogaster*. The group B genes, *Dichaete *and *SoxNeuro *(*SoxN*) have already been well described in the literature [26-28]. Two other group B gene fragments have been identified [25], *Sox21a *and *Sox21b*, but their structure and genomic organisation have not been reported. Using a combination of database searching and DNA sequencing we characterised both of these genes in detail. We find no evidence for any other group B genes in Release 3.2 or Release 4 of the *Drosophila *genome sequence, indicating that there are a total of four in the *D. melanogaster *genome.

### Sox21a

Blast searches of the *Drosophila *genome identified a group B HMG-domain interrupted by a 1655-bp intron in the 70D region of chromosome arm *3L*. Using primers designed against each of these predicted exons we amplified a fragment of 1238 bp from the LD cDNA library produced by the BDGP [29]. For reasons that are as yet unclear, we have been unable to recover a clone from this or any of the several other cDNA libraries that we have screened. The fragment amplified from the library was sequenced in its entirety and found to contain a long open reading frame encoding a 389 amino acid Sox domain protein. The predicted polypeptide initiates with a methionine and probably contains the entire coding sequence for the gene. When aligned with the genome sequence we predict a gene with two exons spanning 2.8 kb. Blast searches with the predicted protein find over 90% identity with a range of group B Sox proteins in the HMG DNA-binding domain. The best scores are with the DNA-binding domains of the vertebrate Sox21 and Sox14 proteins; however, there is little significant similarity outside of the DNA-binding domain. The *Sox21a *gene has previously been reported as *SoxB2-3 *(*CG7345*) and it has been suggested that it may represent a pseudogene [25]. As we show below, RT-PCR and *in situ *hybridisation studies indicate that *Sox21a *is expressed in both *D. melanogaster *and *D. pseudoobscura *indicating that it is not a pseudogene.

### Sox21b

Along with *Sox21a*, Blast searches indicated a second interrupted Sox domain in the same region of the genome. In this case, database searches found a potential cDNA clone from the BDGP (GH07353), which was obtained and sequenced in its entirety. The sequence of the clone revealed a long open reading frame, initiating with a methionine, encoding a predicted polypeptide of 571 amino acids. Alignment with the genome sequence indicates that the gene spans 19 kb of genomic DNA and is composed of 7 exons, the first of which is non-coding. The DNA-binding domain contains two introns; the first, 6388 bp in size, is in the middle of the DNA-binding domain and the second, of 59 bp, is in the same position and frame as the *Sox21a *intron described above. Blast searches with the predicted amino acid sequence find greater than 90% amino acid identity the DNA-binding domains of group B Sox proteins, the highest scores being with *Dichaete*. The sequence indicates that *Sox21b *corresponds to the *SoxB2-2 *gene fragment previously reported [25] and whole mount *in situ *hybridisation with probes derived from genomic DNA and the cDNA clone confirm the pattern of expression previously reported (Figure 2). Thus, both *Sox21a *and *Sox21b *are expressed group B *Sox *genes that have their DNA binding domains interrupted by introns.

**Figure 1:**
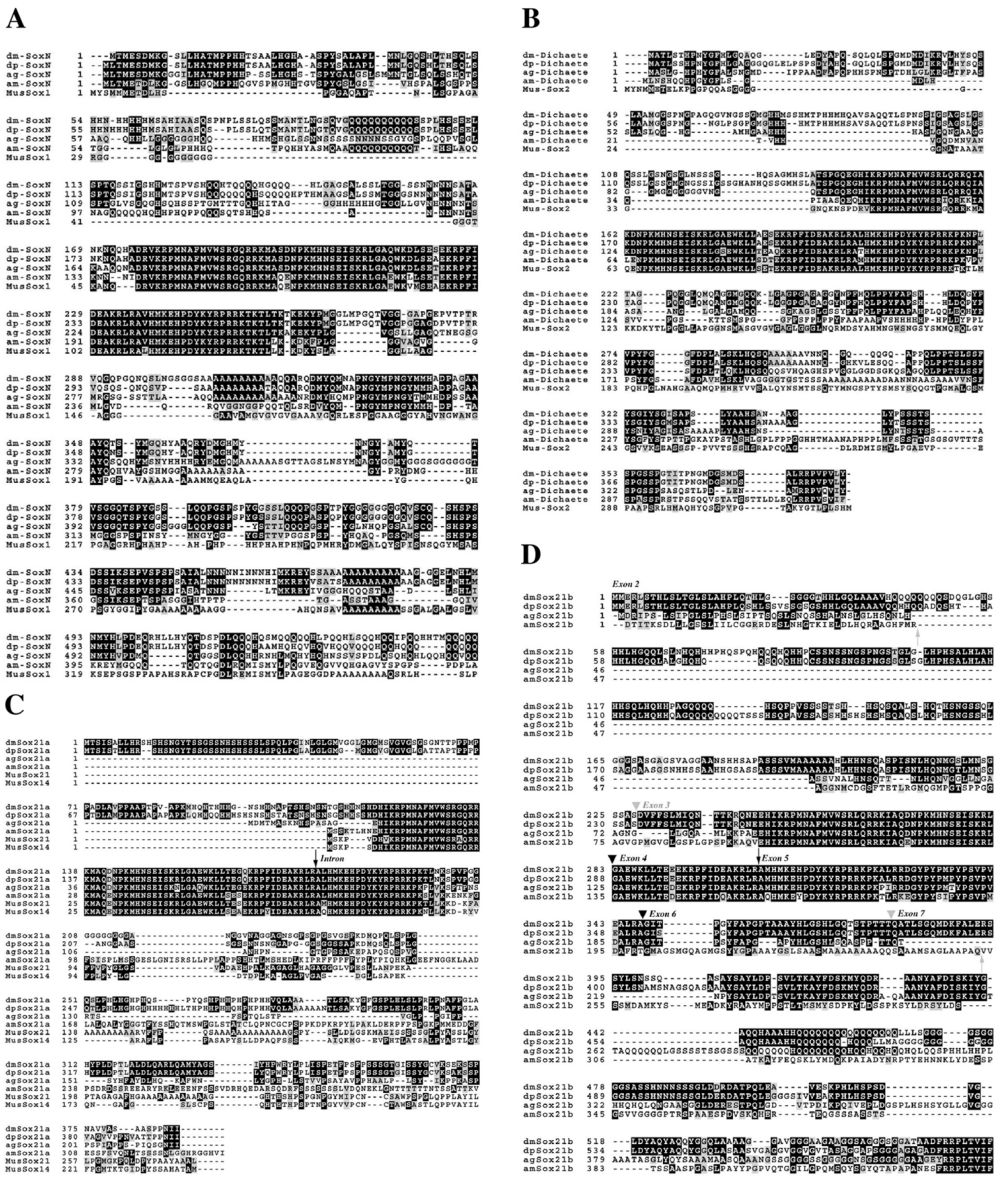
Full alignments of the insect Group B protein sequences. **A) **SoxN. **B) **Dichaete. **C) **Sox21a, the position of the conserved intron is indicated with an arrow. **D) **Sox21b, the location of the exons in the *D. melanogaster *sequence in indicated in italics above the alignment. Black arrowheads above the alignment indicate positions of introns conserved in all four species. The grey arrowheads indicate intron positions conserved in the diptera. The black arrow above the alignment indicates the *drosophila *specific intron and the grey arrows below the alignment indicates the apis-specific introns.

**Figure 2:**
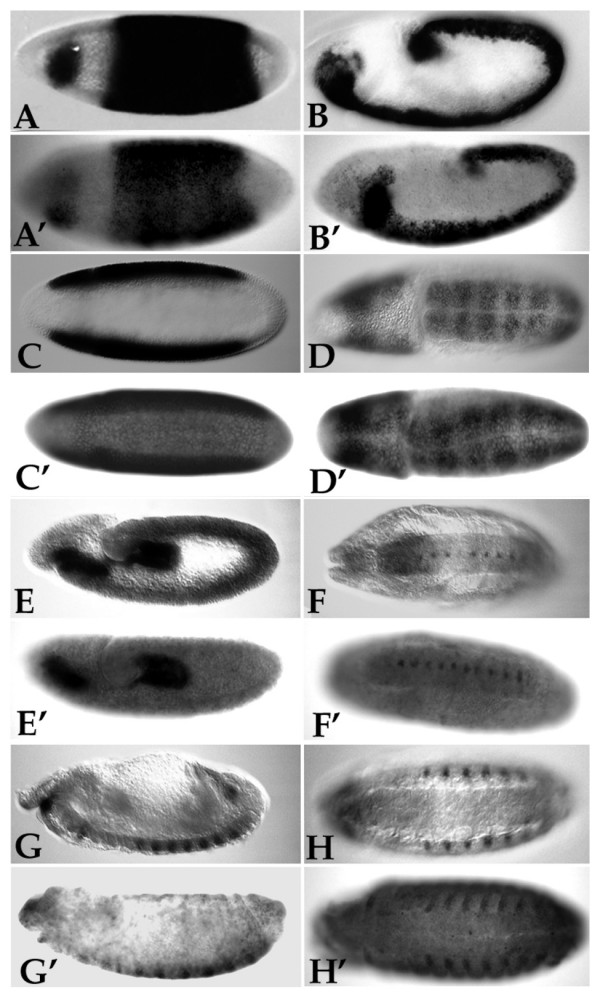
Embryonic expression of group B genes in *D. melanogaster *and *D. *pseudoobscura. A-H, *D. melanogaster*. A'-H', *D. pseudoobscura*; anterior is to the left in all cases. **A-A') **Lateral view of stage 5 embryos showing expression of *Dichaete *in a central domain and the cephalic neuroectoderm. **B-B') **Lateral views of stage 8 embryos showing extensive *Dichaete *expression in the developing CNS. **C-C') **Ventral view of stage 5 embryos showing *SoxN *expression is restricted from the presumptive mesoderm. **D-D') **Dorsal view of stage 8 embryos showing SoxN expression in the CNS. **E-E') **Lateral view of stage 9 embryos showing *Sox21a *expression in the anlage of the foregut and hindgut. **F-F') **Ventral views of stage 14 embryos showing *Sox21a *expression restricted to specific cells in the midline. **G-G') **Lateral views of stage 13 embryos showing *Sox21b *expression in abdominal epidermal stripes. **H-H') **Ventral view of stage 14 embryos showing *Sox21b *expression in abdominal epidermal stripes.

To verify the gene predictions and gain some insight into their possible biological functions we determined the developmental expression of each of the four group B genes by RT-PCR using RNA templates isolated from different stages of the *Drosophila *lifecycle. In the case of *Sox21a *and *Sox21b*, we used primers to the Sox-domain encoding exons spanning a predicted intron. All RT-PCR reactions included a reverse transcriptase minus reaction and the amplified products were verified by sequencing. The results of this analysis are presented in Table 1 and can be summarised as follows: the expression profiles of *Dichaete *and *SoxN *are very similar, they are expressed during embryonic, larval and pupal stages of development, the level of expression reducing during the later stages of pupal development. Both genes are expressed in adult male as well as female flies, with bodies showing stronger expression than heads. *Sox21a *is expressed throughout development and in adults it is stronger in heads than in bodies. In contrast to the other group B genes, *Sox21b *has a more complex expression pattern during development. It is strongly expressed in embryos but is below detectable levels for much of larval and pupal life. After eclosion it is weakly expressed in male heads but not bodies. Thus, in common with mammalian group B genes, all four *D. melanogaster *group B *Sox *are expressed during embryogenesis and at other stages throughout development.

**Table 1:** representation of *Sox *expression in during *Drosophila *development assayed by RT-PCR.

	***Dichaete***	***SoxNeuro***	***sox21a***	***sox21b***
Embryo	+	+	+	+
1^st ^instar	+	+	(+)	(+)
2^nd ^instar	+	+	(+)	-
Early 3^rd ^instar	+	+	(+)	-
Late 3^rd ^instar	+	+	+	-
Prepupa	+	+	+	-
12 h pupa	+	+	+	-
36 h pupa	(+)	(+)	+	-
Heads	(+)	(+)	+	(+)
Bodies	+	+	(+)	-
Male	+	+	+	(+)
Female	+	+	+	-

+ = expression, - = no expression, (+) = weak expression.

#### Group B genes in other insects

Our findings show that *Drosophila melanogaster *has four group B *Sox *genes compared to the five found in vertebrates and, unlike vertebrates, two of the genes contain introns. To investigate whether this particular organization is unique to *D. melanogaster *we searched the available genome sequence of other insects to find potential *Sox *domain genes. Using the *Dichaete *DNA-binding domain as a query, we searched the *Drosophila pseudoobscura, Anopheles gambiae *and *Apis mellifera *genome and EST sequence databases using Blast-P and Blast-N (see materials and methods for EST and genome scaffold accessions). In all three cases we found evidence for four Group B genes and were able to build gene models, from the genome sequence alone or with the addition of EST data where available. The initial characterization of the insect group B genes, based on the HMG-domain sequence, suggests that there is a single orthologue of each *Drosophila *gene in the other three species.

### SoxN

The alignment presented in Figure 1a. shows the similarity between the insect SoxN proteins and mouse Sox1. As previously reported, conservation between vertebrate and invertebrate Sox proteins is mostly restricted to the DNA-binding domains [26,27]. Between the insect proteins there are more extensive regions of homology outwith the DNA-binding domain. The *Drosophila *SoxN sequences show over 90% sequence identity over their entire length and, as expected from the phylogenies based on rDNA and protein coding sequences, the other insect sequences are more diverged [18,30]. *A. gambiae *is overall 64% identical to the *melanogaster *sequence with particularly well conserved regions in the N-terminal 50 amino acids and more patchy conservation C-terminal to the DNA-binding domain. *A. mellifera *is further diverged (52% identity with *Drosophila*). Conserved regions outside the DNA-binding domains among all four sequences are restricted to a stretch of amino acids C-terminal that may represent conserved functional motifs important in transcriptional regulation.

### Dichaete

The situation with Dichaete is similar to that observed with SoxN, and the figures for amino acid identity are virtually identical (Figure 1b). Outside of the DNA-binding domain the Dichaete sequences show even less similarity comparing the *Drosophila *species and the other two insects; conservation between all four being restricted to limited regions C-terminal to the DNA-binding domain. Interestingly, we have shown that the C-terminal region of *D. melanogaster *Dichaete contains sequences required for activity in a context-specific manner [20] and C-terminal regions of the mouse and chicken Sox2 protein are believed to be involved in aspects of correct Sox2 function [31].

### Sox21a

This gene is the least conserved between the four species and outside of the DNA-binding domain they show little similarity with vertebrate group B2 proteins (Figure 1c). There is extensive homology between the two *Drosophila *species, however, the *Anopheles *and *Apis *sequences are very diverged outside of the DNA binding domain. As with *D. melanogaster*, there are no EST sequences available that support the structure of *Sox21a *in the other insects.

### Sox21b

The predicted *Drosophila Sox21b *proteins are again very similar, over 88% identical over their length. The other insect sequences are less well conserved, although the *Anopheles *sequence has a block of conservation C-terminal to the DNA-binding domain, including a Glutamic acid-rich domain (Figure 1d). The predicted *Apis *sequence is less well conserved, we note, however, that all four proteins are identical at the extreme C-terminus. With both the *Anopheles *and *Apis *proteins we cannot confidently predict the N-terminal exons and are unable to find any regions with amino acid similarity to the first 2 coding exons of the *Drosophila *sequences in the *Anopheles *or *Apis *genomic sequence between the end of *Dichaete *and the *Sox21b *Sox-domain encoding exons. Our current models are, however, supported by the available EST sequences for both species although the EST sequences are not full-length. Therefore, the definitive structure of these two insect *Sox21b *genes will require further investigation. Nevertheless, it is clear from the available sequence that orthologues of *Sox21b *are present in other insects.

To confirm the identification of four group B genes in both *D. melanogaster *and *D. pseudoobscura*, we performed whole-mount *in situ *hybridization to embryos of both species using exon-specific probes generated by PCR from genomic DNA. In all four cases we find very similar patterns of expression during embryogenesis. In the case of *Dichaete*, we find blastoderm expression including a broad central domain and a region of expression in the cephalic neuroectoderm (Figure 2A and 2A'). After gastrulation there is extensive expression in the developing CNS (Figure 2B and 2B') including the midline (not shown). With *SoxN *we find conserved blastoderm expression, including an identical restriction from the ventral region of the embryo, followed by extensive expression throughout the developing CNS (Figure 2C to 2D'). With *Sox21a*, we identified conserved expression in the anlage of the foregut and hindgut at stage 12 (Figure 2E and 2E') with later expression in specific cells of the midline after stage 14 (Figure 2F and 2F'). *Sox21b *shows conserved expression in abdominal epidermal stripes from stage 13 (Figure 2G to 2H'). These observations indicate that all four group B genes have conserved expression patterns during embryogenesis.

#### Genomic organisation of group B genes in *Drosophila*: the Dichaete complex

In some vertebrates the two classes of group B genes, B1 and B2, are linked on the same chromosome. In contrast, with *Drosophila *a single gene, *SoxN*, maps to the second chromosome and the remaining three all map to chromosome 3. We examined the organisation of the group B genes in the other insect genomes and found that the situation was very similar to that observed in *Drosophila*. In *melanogaster*, *SoxN *is intronless and sits alone in the middle of an 80 Kb island with no flanking genes for 35 Kb proximal and 45 Kb distal, an unusual organisation for a *Drosophila *gene. We have previously shown that *Dichaete *is controlled by extensive 3' regulatory sequences, suggesting that perhaps the paucity of genes flanking *SoxN *may also indicate the presence of extensive regulatory sequences. In support of this, we find several clusters of predicted transcription factor binding sites from 35 kb upstream to 20 kb downstream of *SoxN *when we use a stringent search criteria with *Cis*Analyst analysis software [32,33] (data not shown). Similar searches with *Dichaete *find previously identified regulatory sequences, suggesting that *SoxN *may indeed be subject to complex regulation. Comparative analysis of the *melanogaster *and *pseudoobscura *genomes with the *Vista *genome alignment viewer [34,35] indicates that the genomic organization is very similar in the two species. The Ensemble annotation of the *Anophelese *genome indicates that the region around *SoxN *is also sparsely populated, with only 2 short stretches of EST homology in the 150 kb flanking *SoxN*. Therefore, it is possible that *SoxN *is subject to complex regulatory control in *Anopheles*. There is currently insufficient contiguous genomic sequence from *Apis *to assess the organization of the *SoxN *region.

**Figure 3:**
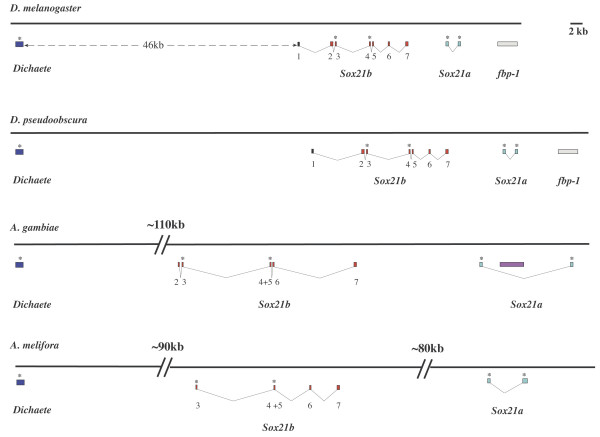
The genomic organisation of the insect *Dichaete *regions. Exons are represented by shaded boxes and introns by the linking lines. A scale bar of 2 kb is indicated. The *melanogaster *and *pseudoobscura *sequences are to scale, the larger distance between *Dichaete *and *Sox21b *in *Anopheles *and *Apis *is indicated by a break in the line, the remainder of the diagram is to scale.

In the 70D region of *Drosophila melanogaster *chromosome arm *3L *the remaining three group B genes are clustered within an 77 kb region (Fig 3). As we have previously reported, *Dichaete *is an intronless gene controlled by at least 30 Kb of regulatory sequence 3' to the transcription unit [36]. 16 kb further distal to these regulatory sequences we find the start of *Sox21b *and a further 28 kb distal to this the start of *Sox21a*. The region ends with the *Fat Body Protein 1 *(*Fbp1*) gene 6 kb downstream of *Sox21a*. The genomic organization of *Sox21b *is highly unusual for a group B *Sox *gene. It is split into seven exons, the first of which is non-coding and exons 3, 4 and 5 contain the DNA-binding domain. All of the predicted splice junctions have consensus GT-AG sequences. *Sox21a*, comprises 2 exons, each containing a portion of the DNA-binding domain. As we note above, the position of the intron, which has consensus splice junction sequences, is in the same position as the second DNA-binding domain intron of *Sox21b *(intron 4).

**Figure 4:**
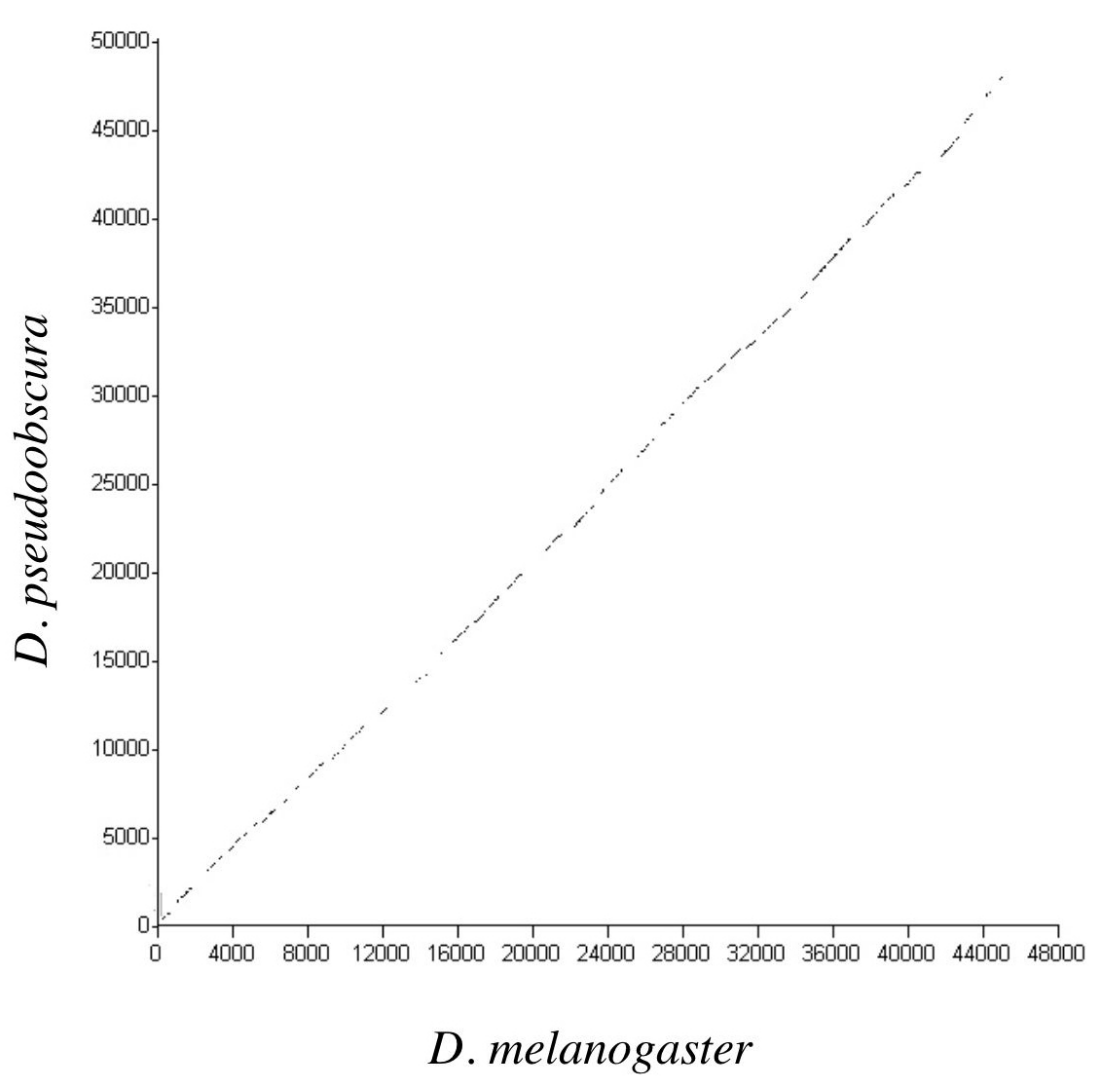
OWEN alignment of the region between *Dichaete *and *Sox21b *in *D. melanogaster *and *D. pseudoobscura *showing extensive sequence similarity throughout the 45 kb region.

In the case of *D. pseudoobscura*, the homology extends from upstream of the *Dichaete *coding region to at least the *Fat body protein 1 *gene downstream of *Sox21a*. The organization of the three *Sox *genes is virtually identical comparing the two species and we could construct gene models including all of the *Sox21a *and *Sox21b *exons. There is absolute conservation of the intron position between both *Drosophila *species, furthermore, the sizes of the introns is also similar, although nucleotide similarity is lower than in coding sequences ranging from 40 – 75%. As with *melanogaster*, we find no evidence for additional genes in the intergenic region between *Dichaete *and *Sox21b*. We used the OWEN sequence alignment programme to plot the conservation between the *Dichaete *– *Sox21b *intergenic region in both species (Figure 4). Throughout the entire region we see that there is a high degree of sequence conservation, since we know that at least 30 kb of this region contains essential *Dichaete *regulatory sequences in *melanogaster*, we predict that regulation in the region will be similar in both species. A suggestion supported by the *in situ *hybridization data presented above (Figure 2).

The organization of the *Dichaete *region in the *Anopheles *genome is very similar to that in the *Drosophila *species with three genes found in a 190 kb region of chromosome arm *3L*. *Dichaete *is intronless and *Sox21b *is located approximately 110 kb downstream of this. There are no other predicted genes in the region. The *Sox21b *has a similar structure to those of the *Drosophilids*, however, it is not identical. We have been unable to find a 5' non-coding exon and, as we note above, the second intron found in the DNA-binding domains of the *Drosophila Sox21b *genes is absent in *Anopheles *with exons 4 and 5 fused. The other introns are, however, conserved in position (figure 1d). With the *Anopheles Sox21a *gene, the single intron position is conserved with the *Drosophila *species, however, the intron is considerably larger and contains an insertion of a Q-class retrotransposon in the sequenced strain [37]. We find no evidence for an *Fbp1 *orthologue in the vicinity, the nearest similar sequence being some 5 Mb away on the same chromosome arm.

The available sequence in the region is more fragmentary in the case of *Apis*. Here we find an intronless *Dichaete *gene and can define two sets of exons corresponding to the split DNA binding domains of *Sox21a *and *Sox21b*. Overall, the organization is similar to the other insects; like *Anopheles*, the intergenic region between *Dichaete *and *Sox21b *is large (~90 kb), however, unlike the other insects the distance between *Sox21b *and *Sox21a *is also large (~80 kb). In the case of *Sox21b *we have used EST sequence to support the gene model we have derived. The EST confirms the first four exons and we predict the terminal exon on the basis of homology with the other species, particularly the terminal 30 amino acids. As with *Anopheles*, the *Apis Sox21b *gene has a single DNA-binding domain intron in the same position of the first *Drosophila *DNA-binding domain intron. The intron immediately downstream of the DNA-binding domain is also conserved in all four insects, however, the remaining two intron positions differ between *Apis *and the other insects. Although the *Apis *assembly is preliminary in this region, with several gaps still present in the sequence, the fact that the gene models are very similar to the other insects and that *Dichaete *and *Sox21b *predictions are supported by EST data suggests that the gene models we propose are likely to be accurate for the majority of the coding sequence.

We compared the *Dichaete *to *Sox21b *intergenic regions of *Anopheles *and *Apis *to the *melanogaster *sequence with the OWEN alignment tool and failed to detect any significant stretches of similarity, even at relatively low stringency. This suggests that if there is conservation in gene regulatory sequences between these diverged insects it may be difficult to detect or have undergone extensive rearrangement.

#### Evolutionary perspective on insect group B genes

**Figure 5:**
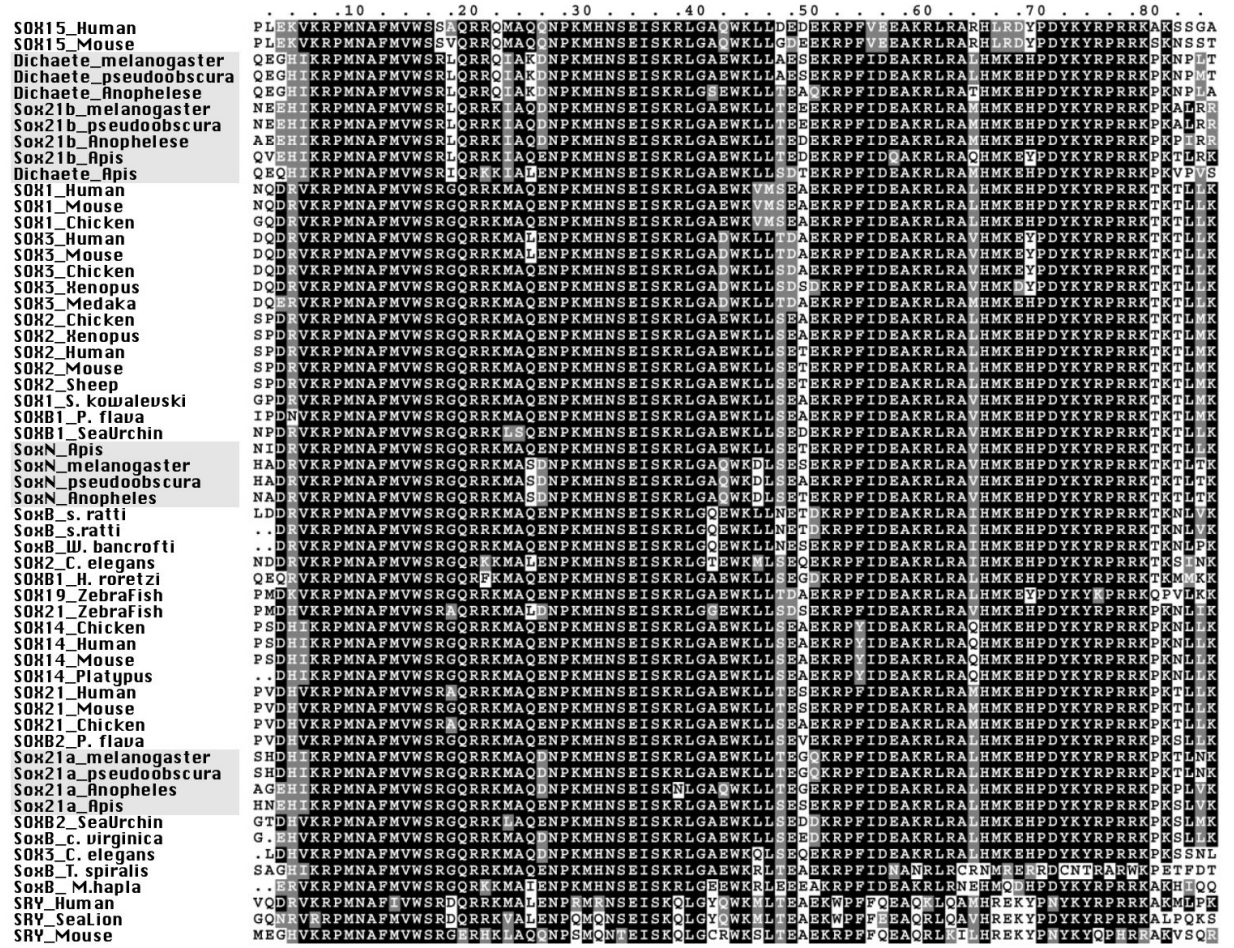
Group B Sox-domain alignment. Clustal X alignment of DNA-binding domain sequences from the insect proteins and representative group B proteins from other species. The insect sequences are highlighted in grey. Accession numbers of protein sequences are as follows: SOX15 Human, O60248; SOX15 Mouse, P43267; Dichaete *melanogaster*, Q24533; Dichaete *pseudoobscura*, TR; Dichaete *Anophelese*, TR; Sox21b *melanogaster*, Q9VUD3; Sox21b *pseudoobscura*, TR; Sox21b *Anophelese*, TR; Sox21b *Apis*, TR; Dichaete *Apis*, TR; SOX1 Human, O00570; SOX1 Mouse, P53783; SOX1 Chicken, O57401; SOX3 Human, P41225; SOX3 Mouse, P53784; SOX3 Chicken, P48433; SOX3 *Xenopus*, P55863; SOX3 Medaka, Q9PT76; SOX2 Chicken, P48430; SOX2 *Xenopus*, O42569; SOX2 Human, P48431; SOX2 Mouse, P48432; SOX2 Sheep, P54231; SOX1 *S. kowalevski*, Q7YTD4; SOXB1 *P. flava*, (Taguchi *et. al. *2002); SOXB1 Sea Urchin, Q9Y0D7; SoxN *Apis*, TR; SoxN *melanogaster*, Q9U1H5; SoxN *pseudoobscura*, TR; SoxN *Anopheles*, TR; SoxB *s. ratti*1, BI323817; SoxB *s. ratti*2, BI323817; SoxB *W. bancrofti*, CD455919; SOX2 *C. elegans*, Q21305; SOXB1 *H. roretzi*, Q86SB8; SOX19 Zebra Fish, P47792; SOX21 Zebra Fish, Q9YH21; SOX14 Chicken, Q9W7R6; SOX14 Human, O95416; SOX14 Mouse, Q04892; SOX14 Platypus, Q8MIP4; SOX21 Human, Q9Y651; SOX21 Mouse, Q811W0; SOX21 Chicken, Q9W7R5; SOXB2 *P. flava*, (Taguchi *et. al. *2002); Sox21a *melanogaster*, Q9VUD1; Sox21a *pseudoobscura*, TR; Sox21a *Anopheles*, TR; Sox21a *Apis*, TR; SOXB2 Sea Urchin, Q9Y0D8; SoxB *C. virginica*, CD648628; SOX3 *C. elegans*, Q20201; SoxB *T. spiralis*, BG302262; SoxB *M. hapla*, BU095063; SRY Human, Q05066; SRY Sea Lion, AAR10360; SRY Mouse, Q05738. TR = This report.

To attempt a classification of the insect group B *Sox *genes, we performed a multiple sequence alignment with the DNA-binding domains of the predicted proteins along with representative group B-like sequences from other organisms (Figure 5). The aligned ClustalX output suggests that the insect Sox domains may be subdivided into 3 classes. The first clearly groups the SoxN proteins from each of the insects with the mammalian Sox1, 2 and 3 proteins. Along with these we find representative sequences from nematodes (*C. elegans*, *S. ratti*, and *W. bancrofti*), hemichordate Acorn worms (*S. kowalevski *and *P. flava*) and the sea squirt (*H. roretzi*). Thus, together these are likely to represent a single class, orthologous to vertebrate group B1 proteins. The second class, the Sox21a proteins, have sequences similar to the mammalian group B2 proteins, Sox14 and 21 and may represent an insect group B2 protein. The third class, containing *Dichaete *and *Sox21b*, are clearly differentiated from all other group B proteins by the presence of a Leucine/Isoleucine residue at position 18, an Isoleucine residue at position 23 and a divergent set of C-terminal amino acids. These two insect proteins may represent an insect-specific group B family. This suggests that a single group B1 protein, represented by SoxN-Sox3 like sequences, was present in a common ancestor before the divergence of vertebrates and invertebrates. Similarly, the close association of the insect Sox21a proteins with nematode and vertebrate Sox14 proteins suggests that these were also present in a common ancestor. The alignments clearly highlight the distinction between the Dichaete-Sox21b pair and other group B proteins, emphasizing a distinct evolutionary history for these proteins in the insects.

Taken together, the analysis presented here shows that the genomic organization and sequence of group B *Sox *genes have been conserved during insect evolution. Particularly striking is the clustering of three genes in a small region of the genome. The structure of these genes and their relationship with vertebrate Group B genes suggest that *SoxN *and *Sox21a *are homologous to vertebrate group B1 and B2 genes respectively, whereas *Dichaete *and *Sox21b *may represent insect-specific group B genes.

## Discussion

The sequence alignments of the HMG DNA-binding domains from insect and mammalian group B Sox proteins suggests that the insect proteins may be separated into three distinct groups. The first, containing SoxN, aligns with the vertebrate Sox1, 2 and 3 proteins and most likely represents an orthologue of the vertebrate group B1 class. This conclusion, based on sequence, is supported by the functional analysis of group B1 proteins in vertebrates and *Drosophila*. In both cases, group B1 genes are expressed from the earliest stages of CNS development and are implicated in regulating early neural specification [21,22,38,39]. In addition, we have evidence that mammalian *Sox1 *genes can rescue *SoxN *phenotypes in the *Drosophila *CNS, supporting the view that these proteins are functionally conserved (P. Overton and S.R. unpublished observations). The group B sequences isolated from the basal chordates, acorn worm and sea squirt, have also been shown to be expressed early in the specification of the CNS [40,41]. Thus, it appears that all metazoans studied to date have at least one group B gene with expression marking neural lineages early in development. Further studies of primitive invertebrates will determine whether group B *Sox *expression is a universal marker for CNS development.

In a previously published phylogenetic studies it was suggested that *Dichaete *be classified as a Group B2 protein [3]. However, while the analysis clearly differentiates between the group B proteins and other fly Sox proteins it could not unambiguously resolve the relationship between each of the group B proteins. In terms of function and expression, the *Dichaete *gene behaves very much like a group B1 gene, it is expressed early during CNS development and is required for neural differentiation [20,42]. We have previously shown that the mouse *Sox2 *gene efficiently rescues *Dichaete *phenotypes, further supporting a functionally similarity between *Dichaete *and vertebrate group B1 genes [20,42]. In contrast to the conclusion based on functional studies, the sequence analysis suggests that insect Dichaete DNA-binding domain sequences are markedly different from other group B1 proteins and are more similar to group B2 proteins. The conservation of the insect sequences indicates that a *Dichaete-*like sequence was present at least 300 My years ago, when *Apis *and the Diptera last shared a common ancestor [18]. We believe that the functional evidence is more convincing than the arguments based on sequence alignments and therefore suggest that Dichaete represents a group B1 function that has diverged from the canonical group B1 sequence, presumably due to selection for insect-specific functions. For example, Dichaete is required for early segmentation in the *Drosophila *embryo, a highly derived function, and it may be that sequence changes in the HMG-domain have been selected for such a function while still allowing a role in CNS-specification. As with *Drosophila*, both *Anopheles *and *Apis *are long germ insects that share some aspects of early development such as the early appearance of striped domains of *even skipped *expression [43,44]. Thus it is possible that insect *Dichaete *genes have a common role in early patterning events. It will be of considerable interest to examine the complement of group B *Sox *genes in Coleoptera, Homoptera or Orthoptera to see if the HMG domain sequence and gene organisation is the same as the insects so far sequenced. To investigate this we used the Dichaete DNA-binding domain to search the available sequence of the silk moth *Bombyx mori*. [45] and found a single Group B gene that was clearly an orthologue of the *Dichaete *genes discussed here, containing the diagnostic Leucine and Isoleucine residues described here.

As with vertebrate group B1 genes, *SoxN *and *Dichaete *are expressed in broadly overlapping domains and act partially redundantly in CNS specification [21,22]. The close similarity between the expression and function of *SoxN *and *Dichaete *in the CNS raises the possibility that they arose from a common ancestor by a duplication event and may thus share some common regulatory sequences. However, when we compared the sequences 5' or 3' to *SoxN *with the *Dichaete *3' sequence we could not detect any sequence similarity indicating that any conservation in regulatory sequences is not visible at a large scale; this is not entirely surprising since we cannot detect any sequence similarity between the *Dichaete *regulatory sequences from *Drosophila *and *Anopheles*, while our analysis indicates the divergence of *SoxN *and *Dichaete *predates the *Drosophila*-*Anopheles *divergence.

Based on the sequence alignment of insect Sox21a DNA-binding domains with those of vertebrate Sox14 proteins, it is possible that Sox21a may be an orthologue of the group B2 class. It has been suggested that in chicken Sox14 and Sox21 act as antagonists of group B1 function in a subset of the developing CNS [6]. The function of *Sox21a *in *Drosophila *is not known at present, however, *Sox21a *is expressed late in the development of the embryonic CNS midline, a site of *SoxN *and *Dichaete *expression, indicating there is the potential for the type of antagonistic interaction proposed for vertebrates. The Sox21b DNA-binding domain sequence indicates that it is closely related to Dichaete. Both these proteins have a set of unique residues in their DNA-binding domains that are not found in any other group B proteins identified to date. The *Sox21b *gene is conserved between the insects and its close similarity to *Dichaete *suggests that both genes arose from a common origin in the ancestor of the arthropods after their divergence from the nematodes since there is no close sequence in *C. elegans *or its relatives. In terms of expression, *Sox21b *is expressed in the large hindgut along with Dichaete, supporting the possibility that it may also antagonise the activity of Dichaete. In this respect then *Sox21b *may represent a group B2 function. It is therefore possible that insects contain 2 group B1 class activities, involved in early CNS development, and two B2 class genes. Again we emphasise that the functional assignment of the insect genes may contrast with the data derived from sequence analysis, which predicts a single group B1 gene and three group B2 genes. We suggest that the separation of group B Sox domains into a B1 class and B2 class based solely on sequence does not reflect meaningful functional differences in insects. We have initiated a functional analysis of *Sox21a *and *Sox21b *in the hope that we can clarify this issue.

The genome organisation of the Dichaete cluster is unusual, not only are three genes clustered together in the genome but two of them, *Sox21a *and *Sox21b*, have introns within the HMG-domain. The single *Sox21a *intron is conserved in all four of the insect genes suggesting that it is ancestral to the insects. *Sox21b *is more complex, there are six introns in *melanogaster *and *pseudoobscura*, four of these are conserved in *Anopheles *and two are conserved in *Apis*. In the *Drosophila *species, there are two introns in the DNA-binding domain, the first of which is present in all four insects. The second intron, in an identical location to the *Sox21a *intron, is only found in the two *Drosophila *species. A simple model of a single intron loss is therefore unlikely to account for this since both *Apis *and *Anopheles *do not have the intron. It is possible that *Apis *and *Anophelese *lost the intron independently or, alternatively, that the common ancestor of the *Drosophila *species gained the intron, perhaps via a gene conversion event with *Sox21a*. Interestingly, the two group B genes from *C. elegans *also contain introns in the DNA-binding domain, in identical positions in both genes, but they are in different positions to the *Sox21a *and *Sox21b *introns. This suggests that the common ancestor of insects and nematodes did not contain DNA-binding domain introns and that these have been acquired independently in both lineages.

The conservation of genome structure with the insect *Dichaete *cluster suggests that there may be functional constraints on the organisation. We suggest that this is likely to be a reflection of shared regulatory sequence since the region between *Dichaete *and *Sox21b *in *melanogaster *contains extensive regulatory sequences essential for correct *Dichaete *expression. We note that both *Sox21a *and *Sox21b *have expression domains that overlap with *Dichaete*, in the midline for *Sox21a *and the hindgut with *Sox21b*. These expression domains may therefore be controlled by common regulatory sequences and the need to maintain coordinated regulation of the three genes has maintained the integrity of the cluster in the insects. The conservation in expression between *D. melanogaster *and *D. pseudoobscura *is consistent with this view; it will be of interests to examine the expression of the all of the *Sox *genes in *Anopheles *to further explore this hypothesis.

## Conclusion

**Figure 6:**
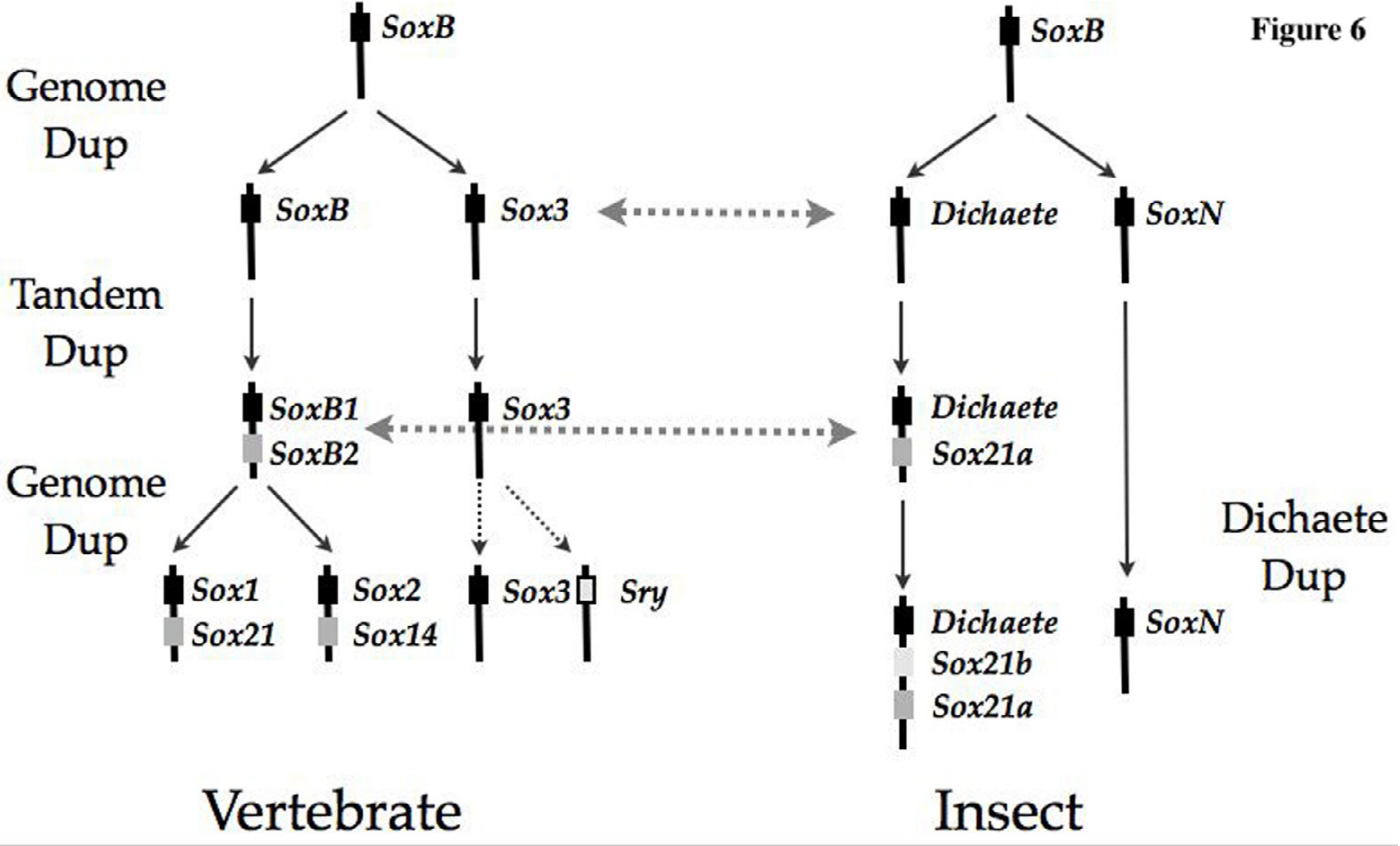
A model for the evolution of Group B *Sox *genes in insects following the proposal of Kirby *et al *(2002) for vertebrates. In this view an ancestral group B gene is duplicated during an ancient genome duplication event to generate *Dichaete *and *SoxN*. A tandem duplication of Dichaete generates *Sox21a*; these events would be common to the ancestor of vertebrates and invertebrates. In insects, a further duplication of *Dichaete *gives rise to *Sox21b*.

Taking our observation together, we propose a simple model for the evolution of group B *SOX *genes (Figure 6). We base our model on the proposal of Kirby *et. al. *[13] who suggest that a single group B gene underwent a duplication to generate two *Sox3- *like genes. We propose that these are represented by *SoxN *and Dichaete in the insects. A further tandem duplication of one of these genes generated linked group B1 and group B2 genes. We propose that this is represented by *Dichaete *duplicating to generate *Sox21a*. *Sox21a *would then acquire the sequence changes characteristic of the group B2 class of proteins. We suggest that these events predate the Protostome-Deuterostome divergence over 650 My ago [46] and provide the basal *Sox *gene complement of the Bilateria of three group B *Sox *genes. After the divergence of the lineages leading to vertebrates and invertebrates, Dichaete diverged from the canonical group B DNA-binding domain sequence and then underwent further duplication event, at least predating the divergence of the holometabolous insects, to generate *Sox21b*. An analysis of the group B family in other insects and basal chordates will be required to definitively describe the ancestral situation.

## Methods

### Genome sequences

The following sources were used to obtain genome sequence: *D. melanogaster *(Release 3.2, [47,48]) from FlyBase [49] and the following scaffolds were used; AE003535 for the *Dichaete *region and AE003622 for the *SoxN *region. *D. pseudoobscura *(Freeze_1 assembly) was obtained from the Human Genome Sequencing Center, Baylor College of Medicine (HGSC-BCM [50]) and the following scaffolds used; Contig5946_Contig6670 for the *Dichaete *region and Contig1741_Contig5707 for the *SoxN *region. *Anopheles gambiae *genome sequence release 19.2a.1, compiled by the International *Anopheles *Genome Project [51], was obtained from the Ensembl server at the Wellcome Trust Sanger Institute [52]. In the Ensemble annotation the *Sox *genes have the following accessions: *SoxN *(ENSANGG00000019842), *Dichaete *(ENSANGG00000010137), *Sox21a *(ENSANGG00000010002) and *Sox21b *(ENSANGG00000009947). *Anopheles *EST sequences representing *Dichaete *(TC44994) and *Sox21b *(TC45155) were obtained from The Institute for Genome Research [53]. *Apis mellifera *Genome assembly Amel_1.1 was obtained from HGSC-BCM [54] and the following scaffolds used: for the *Dichaete *region, Group8.12 (*Dichaete *and *Sox21b*) was found to overlap by 4.5 kb with GroupUn.570 containing *Sox21a *and the sequences were combined into a single contig. *SoxN *was contained within Group17.6. In addition a search of the Honey Bee Brain EST project [55,56] uncovered two EST sequences corresponding to *Dichaete *(BB170009A10D01) and *Sox21b *(BB170011B20A11). These were used to verify the exon predictions from the genome sequence. Vertebrate group B sequences were obtained from Uniprot. Nematode sequences were recovered by Blast searches of the EST collections at Nematode.net (Genome Sequencing Center, Washington University, St Louis, [57]).

### Informatics tools

Homology searching was performed using the Blast algorithm [58] at Sanger, HGSC-BCM and Berkeley Drosophila Genome Project [59] web sites. Genomic sequences were imported into Artemis v5 [60,61] and annotated manually using the Blast output as a guide. Multiple Sequence alignments were performed locally using ClustalXv1.8 [62] and graphically represented with BoxShade [63]. The alignment of intergenic regions was performed with OWEN [64].

### Molecular biology

A cDNA clone for *Sox21b *(GH07353) was obtained from the *Drosophila *gene collection [29] and sequenced on both strands using an ABI prism kit in the Genetics Department sequencing core. PCR and RT-PCR amplifications were carried out using minor modifications to standard techniques [65] using the following primer combinations:

*Melanogaster *primers for RT-PCR

Dichaete F ACAATCCATTCCATCAACTACC

Dichaete R TTGGTGTTCCCTCCTTACTC

Sox21B F AGTCTCATGAACAGCGGAAG

Sox21B R GGAGTTGCTCAGATACGACG

SoxN F CAGCAGCAACAGCAACACTAC

SoxN R TTTCATCGCCTCGCCACAAC

*Pseudoobscura *primers for *in situ *probes:

Dp-Dichaete F CGAACTACGGATTCCACCT

Dp-Dichaete R CATTCCGTTGGCCTGCAT

Dp-SoxN F AGCTGAGTCACCATAACCAC

Dp-soxN R GTCATGTGATGGCTACCAA

Dp-Sox21A Exon1 F GAGCATCTCGACGCTACTAC

Dp-Sox21A Exon 1 R GGAATTGGAGTGGCTATGAT

Dp-Sox21A Exon 2 F CTAAGGACATGCAGTCACAG

Dp-Sox21A Exon 2 R GACTTCACGCAGCCGTAGGAT

Dp-Sox21B F CGTCTATCCACACACCTGTC

Dp-Sox21B R GACGATGTCTGCTGCTGTT

Whole-mount *in situ *hybridisation to *Drosophila *embryos was performed using minor modifications to a standard protocol [66].

All genetic nomenclature is according to FlyBase [49].

## Authors' contributions

C.McK. performed the sequencing, mapping of the *Drosophila Sox21a *and *Sox21b *genes in and the *in situ *hybridisation experiments. G.W. carried out the RT-PCR analysis. S.R. designed the experiments, carried out the genomic analysis and wrote the paper.
